# Ethics and governance of trustworthy medical artificial intelligence

**DOI:** 10.1186/s12911-023-02103-9

**Published:** 2023-01-13

**Authors:** Jie Zhang, Zong-ming Zhang

**Affiliations:** 1 Institute of Literature in Chinese Medicine, Nanjing University of Chinese Medicine, Nanjing, 210023 China; 2 Nantong University Xinglin College, Nantong, 226236 China; 3 Research Center of Chinese Medicine Culture, Nanjing University of Chinese Medicine, Nanjing, 210023 China

**Keywords:** Artificial intelligence, Healthcare, Ethics, Governance, Regulation, Data, Algorithms, Responsibility attribution

## Abstract

**Background:**

The growing application of artificial intelligence (AI) in healthcare has brought technological breakthroughs to traditional diagnosis and treatment, but it is accompanied by many risks and challenges. These adverse effects are also seen as ethical issues and affect trustworthiness in medical AI and need to be managed through identification, prognosis and monitoring.

**Methods:**

We adopted a multidisciplinary approach and summarized five subjects that influence the trustworthiness of medical AI: data quality, algorithmic bias, opacity, safety and security, and responsibility attribution, and discussed these factors from the perspectives of technology, law, and healthcare stakeholders and institutions. The ethical framework of ethical values-ethical principles-ethical norms is used to propose corresponding ethical governance countermeasures for trustworthy medical AI from the ethical, legal, and regulatory aspects.

**Results:**

Medical data are primarily unstructured, lacking uniform and standardized annotation, and data quality will directly affect the quality of medical AI algorithm models. Algorithmic bias can affect AI clinical predictions and exacerbate health disparities. The opacity of algorithms affects patients’ and doctors’ trust in medical AI, and algorithmic errors or security vulnerabilities can pose significant risks and harm to patients. The involvement of medical AI in clinical practices may threaten doctors ‘and patients’ autonomy and dignity. When accidents occur with medical AI, the responsibility attribution is not clear. All these factors affect people’s trust in medical AI.

**Conclusions:**

In order to make medical AI trustworthy, at the ethical level, the ethical value orientation of promoting human health should first and foremost be considered as the top-level design. At the legal level, current medical AI does not have moral status and humans remain the duty bearers. At the regulatory level, strengthening data quality management, improving algorithm transparency and traceability to reduce algorithm bias, and regulating and reviewing the whole process of the AI industry to control risks are proposed. It is also necessary to encourage multiple parties to discuss and assess AI risks and social impacts, and to strengthen international cooperation and communication.

## Background

Artificial intelligence (AI) has been described as the fourth industrial revolution following the first “steam engine revolution”, the second “electrical revolution”, and the third “digital revolution” [1]. From autonomous vehicles to virtual assistants and software for translation, AI is being used in a wide range of scenarios in different fields, and the medical field is also undergoing a significant transformation. With the accumulation of big data in medicine and health, AI is becoming more and more implementable in healthcare. From early rule-based algorithms to machine learning to deep learning, medical AI is currently used in various medical fields such as medical image analysis, disease screening and prediction, clinical decision support, surgical robotics, health management, virtual medical assistants, and aiding in screening drug targets [2–5]. The commercial value is also continuously driving the technological innovation in medical AI. The health AI market is estimated to grow tenfold from 2020 to 2026, reaching $45.2 billion in 2026 [6] attracting technology giants not traditionally associated with health, such as Google, IBM and Microsoft, to join and dominate the market [7].

The rapid development and application of medical AI symbolize more universal and efficient medical assistance, more convenient and accurate medical treatment, bringing a revolutionary breakthrough in traditional medicine at many levels. However, most of the current medical AI does not replace human doctors but rather speeds up and helps humans to diagnose [8] with the final decision still coming from humans [9] which is also known as “Augmented Intelligence” [10]. In the future, medical AI may fundamentally change the medical model and decision-making mode [11, 12] and even replace humans or surpass them.

With the rapid development of technology, people increasingly find that while enjoying the convenience of science and technology, there are also various uncertainties and uneasiness, which brings people confusion and a sense of crisis about the development of technology. The social consequences of technology are difficult to predict early in its development. When the demand for technological change becomes intense, such change has become very difficult and time-consuming. It is called the “dilemma of technology control”, which is also known as the “Collingridge dilemma” [13]. Technological breakthroughs tend to inflate desires rather than make people more rational. By the time we know the consequences of the technology, our ability to control it becomes extremely limited, because the technology has gained enough momentum and has its own path. If the new technology leads to an unexpected deterioration, it will be, as Jesus said: the end. We need to prejudge and plan ahead to get out of the dilemma.

The rapid development of AI technology is also accompanied by many risks and challenges. There is now a growing consensus among experts to view the adverse effects of AI as ethical risks [14]. These adverse effects influence the attitudes of the public and health practitioners towards medical AI. AI companies try to convince people that their behaviors, products, and services are ethical by “Ethics Bluewashing”, but it is not the case [15]. They also try to avoid ethical discussions by discussing “trustworthy”, because trustworthiness seems to be described by degrees, such as a product or service being more trustworthy than others. As for trust, Simpson [16] believed that trust arises from the fact that we rely on the cooperative behavior of others, and sometimes, we rely not on other people, but on things. While things are not capable of cooperative action, whether they will prove to be reliable may be opaque to us, and their unreliability with connotations of exposure to risk and uncertainty of outcome may affect our trust, which is called predictive trust. As such, discussions on trustworthy AI are related to ethical risks. The ethics of a technology directly impinge on the trust in that technology through the moral element of trust.

This paper focuses on trustworthy medical AI from an ethical perspective. We analyzed both the design level (whether the technology is reliable) and the application level (the human impact of the use of medical AI) to assess the factors that affect people's trust in medical AI, and proposed corresponding governance countermeasures from ethical, legal and regulatory aspects according to the ethical governance framework, which points out the direction for the controllable and sustainable development of medical AI.

## Method

**Fig. 1:**
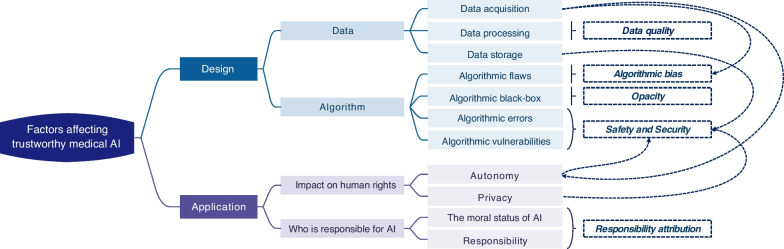
Factors affecting trustworthy medical AI

The main factors affecting the trustworthiness of medical AI include whether it is technically safe and reliable, and whether it is used in a way that respects fundamental human rights and conforms to universal human values. We adopted a multidisciplinary approach to analyze the factors affecting the trustworthiness of medical AI from both the design and application levels (Fig. 1). The design level is whether the technology is reliable and the application level is the impact on humans when using medical AI. In the design of medical AI, the safety and reliability of the technology mainly come from data and algorithms aspects. The data aspect includes data acquisition, processing and storage, involving issues such as informed consent, data quality and privacy protection. The algorithm aspect includes algorithmic flaws, algorithmic black-box, algorithmic errors and algorithmic vulnerabilities, involving issues such as algorithmic bias, safety and security, and opacity. In the application of medical AI, the impact on human rights and the attribution of responsibility affect the trustworthiness of medical AI. The impact on human rights mainly involves human autonomy and privacy, while the attribution of responsibility mainly related to the moral status of AI and who is responsible for AI. We analyzed these issues involved and refined five subjects that affect the trustworthiness of medical AI: data quality, algorithmic bias, opacity, safety and security, and responsibility attribution. These subjects were discussed from the perspectives of technology, law, and healthcare stakeholders and institutions. Subsequently, we proposed corresponding countermeasures for trustworthy medical AI from the ethical, legal and regulatory aspects following the ethical governance framework of ethical values-ethical principles-ethical norms.

## Results

### Data quality

In the era of big data, the data are numerous and complicated. There is a saying in the computer field, “garbage in, garbage out.” The quality of data directly determines the quality of medical AI. Medical-related data mainly comes from multi-source and heterogeneous data including literature data, clinical trial data, real-world data, and also health data collected by a large number of intelligent wear, fitness applications and other devices [17]. AI systems are only as good as the data provided to them [18]. It is reported that IBM’s Watson for oncology system often recommends "unsafe and incorrect" cancer treatments, because the data used to train Watson's algorithm are not from real patients but hypothetical and insufficient data from virtual patients. The treatment recommendations were derived from a few experts in each type of cancer, rather than from relevant guidelines or reliable evidence [19]. These data do not represent the actual, complex clinical situation, which will inevitably affect the accuracy and generalizability of the algorithmic model.

Ensuring high-quality data is the primary prerequisite for AI development. At present, there are some problems in the data of medical AI, such as data errors and omissions in the original data entry process, lack of unified metadata standards, difficulties in data fusion, lack of data management, non-standardized strategy for data cleaning, and much medical data is stored in unstructured forms such as text and images, which increases the difficulty of data management and integration. The accuracy of the data annotation determines the quality of the dataset. Even if the data is accurate and representative, the result will be meaningless if there is a problem in the data annotation process. The risk in the data annotation is mainly in the consistency aspect. For example, the gold standard for clinical diagnosis of pulmonary nodules is a pathological biopsy, but not every patient with lung nodules will have a biopsy [20]. CT scans are usually used to screen for lung nodules clinically. The image data of medical AI based on CT imaging of lung nodules need to be annotated by clinically experienced doctors. The CT imaging features of lung nodules include nodule size, density (CT value), nature (solid nodule/glassy nodule), and signs (lobar sign, burr sign, concave umbilical sign, vascular cluster sign) [21]. However, due to the different equipment models of hospitals and software development institutions, doctors’ judgement bias and different standards, quality, and formats for labeling image data, will lead to annotation deviation, resulting in confusion and controversy. AI is also used in traditional Chinese medicine(TCM). TCM doctors have accumulated a large amount of real and practical unorganized clinical cases in their long-term clinical practices, which need to be converted into computer language, the core technology of which is natural language processing (NLP) and medical text recognition. The machine needs to understand the semantics of the text. However, since most of the texts in TCM are unstructured data and Chinese medicine has more non-standardized expressions, manual annotation is extremely costly and difficult, which greatly affects data quality. As can be seen, humans are still widely involved in many aspects of medical AI design and development, and their professional skills, compliance awareness, and moral quality should be reviewed as necessary to control risks.

### Algorithmic bias

There are some who believe that it may be possible for AI to mitigate existing biases in the healthcare system, such as reducing human errors [22] and the cognitive biases of physicians in determining treatment decisions, leading to more equitable outcomes [23]. However, algorithms can also lead to biased results. Bias is often encoded and expressed through machine learning, which can have far-reaching effects on the outcomes [24].

Algorithmic bias includes both human-induced bias and data-induced bias. Human-induced bias is intentionally or unintentionally written by the developers because individuals are always influenced by their own moral perceptions and relevant interests, which affects data training [25, 26]. For example, data labeling (classification), noisy data handling, setting variables and weighting attributes may reflect the designer's biases or limitations. Considering the problem of algorithm bias from the subjective will of the algorithm designer, it is either an unintentional behavior or a specific design under the trade-off of interests, and social bias can be unintentionally reflected or purposefully embedded in the system design by designers to produce output results unfavorable to the disadvantaged people. Not only do algorithms inherit human biases, but such biases are also likely to be reinforced and amplified with the accumulation of data and iterations of algorithms. How to identify and assess the value of designer subjectivity permeating the algorithm may require a longer-term, multi-user or big data-based analytical judgment. Data-induced bias refers to the bias when the training data is not representative or insufficient that affects the algorithm model [27, 28]. For example, if the algorithm is developed with training data that primarily involves Westerners, it may be less accurate in diagnosing Asian people. Similarly, intelligent TCM expert systems based on big data of sutra prescriptions of TCM and a large number of local Chinese samples may not be fully applicable to Westerners. Second, deep learning is a typical “black box”, it is opaque and uninterpretable, which makes the biases difficult to be detected. These biases may be continuously replicated and amplified in algorithm and lead to biased prediction results, which may cause discriminatory treatment of certain people in medical care and even lead to medical safety accidents. For example, an AI for melanoma diagnosis is difficult to apply to darker races because there are few medical images of darker races in the skin lesion database [29]. Another example is an algorithm for health decision-making that incorrectly uses health care costs (rather than disease) to represent the level of health need. Because less money is spent on the health of black people, the algorithm incorrectly concludes that black people are healthier, even though they are much sicker in the datasets [30]. Much of the training data for healthcare AI is sourced from high-level medical facilities, which leads to frequent bias when it is used in lower-level medical facilities such as community clinics [31]. In healthcare, biased algorithms may underestimate or overestimate certain patients’ risks, and unrepresentative data may consolidate or exacerbate health disparities. It follows that data representativeness and diversity are essential, as medical AI must be generalizable and transferable and should be equally applicable to patients across races, geographies, genders, and levels of care. These issues can be addressed by training algorithms from more diverse datasets.

### Opacity

There are three possible reasons for opacity: (1) algorithms are trade secrets that companies intentionally hide; (2) the inability of lay people to understand programming and algorithmic techniques; (3) the complex nature of the algorithms themselves, which are incomprehensible to humans [32]. The first two types of opacity can be improved by establishing better regulation and education. The third type of opacity is inherent to machine learning and is also known as the algorithmic “black box”, meaning that its inputs and outputs are visible and understandable, but the process from input to output cannot be explained or understood. Not all algorithms are black boxes [33]. The opacity problem arises mainly in the second generation of AI, data-based machine learning, which uses large datasets to automatically learn functional associations between predictor variables and outcomes without explicit programming [34]. In contrast to traditional algorithms, machine learning is based on data rather than rules. The form of the function that relates the input data to the output is too complex for non-specialists to understand, and even the designer may not be able to understand the logic of the calculation. For example, AlphaGo is a typical machine learning algorithm, but its developers are also unable to explain its moves and layouts when playing against human Go champions. Deep learning is a newer algorithm of machine learning. Compared with general machine learning, deep learning is a brain-like computing method which imitates the multilayer structure of the human brain neural network and designs multiple hidden layers in the programming to calculate and train in the form of a multilayer neural network. It can realize self-learning and automatic feature extraction without human assistance [34]. For example, AlphaGo Zero uses a deep learning algorithm that does not require human input of large amounts of game data for training like AlphaGo, but starts with a blank board and plays millions of games against itself to reach a level that is much higher than AlphaGo [35]. Deep learning is widely used in medical AI and performs very well currently in medical imaging analysis [36] and clinical risk prediction [37] significantly improving doctors’ diagnostic and predictive capabilities. However, explaining deep learning-based medical AI is almost impossible, which is related to the connection complexity and mathematical abstraction of deep neural networks. People cannot explain how the machine makes judgments [38]. In addition, large algorithms in practice are usually a combination of multiple algorithms, which is beyond the comprehension of human intelligence.

The opacity of the algorithm creates “ignorance” among human agents and will affect the trust of patients and clinicians in AI tools. According to the research, patients are generally repulsed by unexplained AI interventions in diagnostic and treatment sessions. They usually only accept AI to handle administrative matters such as registration, bill payment, and guidance [39]. Moreover, doctors seem to be more repulsed by medical AI than patients. Based on an inherent mindset emphasizing science and logic, doctors are less willing to trust and rely on things they cannot explain [40]. If doctors do not understand why the algorithm made this diagnosis, should they rely on the software? How can they convince patients of the treatment plan? Not to mention what information they should provide to the patients. Taking a step back, even if a medical AI could perform computer and mathematical-level explanations, it would be difficult to translate them into meaningful medical explanations. It is because AI is always a statistical correlation analysis rather than a medical causality analysis [41].

The technical community has proposed the goal of explainable AI (XAI) [42] that is, making AI explainable while maintaining high performance. Scientists have worked hard to open the black box of AI, proposing many paths and methods to crack the black box, such as the twin-systems approach [43] and the dialog model [44] and have achieved some results. For example, Google has recently claimed that they have preliminarily cracked the mechanism of a medical AI for diagnosing eye diseases [45]. However, there is a consensus among computer scientists that an inherent tension is between the performance and explainability of machine learning, with the best-performing algorithms being the least transparent and those that provide precise explanatory algorithms often being less accurate [46] which seems to be a logical paradox. It is foreseeable that the future of interpreting AI algorithms will be a long and challenging task.

### Safety and security

The safety issues of medical AI are the risks and harms that occur in its practices, such as program errors, being affected by cybersecurity, the need for adequate testing, difficult software certification, etc., covering various legal and ethical issues [47]. No technology is 100% safe. However, with medical AI, the first thing that comes to mind is to ensure its safety which is absolutely necessary, because the purpose of medical AI should be dedicated to protecting and promoting human health. When medical AI goes wrong, it can cause serious harm to people. Between 2000 and 2013, surgical robots in the United States were responsible for at least 1,391 harm-causing incidents and 144 deaths [48].

The risks of medical AI comes more from the algorithms. First, the algorithmic black box makes models lack explainability and are difficult to proofread. If the algorithm is flawed or incorrect, the output will lead to even greater errors, which will most likely cause diagnostic errors, harm human health, and even deprive human lives. In 2015, the British used a medical robot to perform heart valve repair surgery, and the robot not only made serious operational errors, but also interfered with the correct operation of human doctors, resulting in the patient's death [49]. It is impossible to develop a code that covers all possibilities. Therefore, safety flaws in AI may endanger more patients than the possibility of a single misdiagnosis by a doctor because automated systems will replicate more errors [50]. In 2019, the U.S. Food and Drug Administration(FDA) urgently recalled Zimmer Biomet’s ROSA Brain 3.0 robotic surgical system due to errors in software that caused the robot's arm to be in the wrong position [51] Second, most current medical AI algorithms are trained using historical data from retrospective studies. When encountering real-world data that differ from that in the training datasets, the performance of AI may be worse, leading to clinical risks [52]. AlphaGo crashed against Lee Jae Suk due to blind spots of algorithms. When AI encounters unimaginable situations, it may follow its instincts and take strange actions. Third, there are also risks associated with the potentially autonomous functions of AI applications. For example, medical chatbots provide diagnostic and treatment recommendations in order to reduce unnecessary doctor visits. However, these medical chatbots may also harm patients if they are not continuously updated, checked, or regulated [7]. There are also potential risks when a care robot is involved in patient and elderly care [53]. When the standard of care for cancer patients changes, such as adjusting medication doses to be more beneficial for a specific patient, and the care robot does not keep the information up to date, this can do harm to the patient's health [54]. Moreover, due to the limitation of the built-in program algorithm, the care robot may restrict the patient's autonomy, such as limiting the patient's movement to protect their safety. They may also violate the patient's privacy. Doctors and family members may know behaviors that patients do not want to be known by others, such as changing clothes, bathing, etc., when using the care robot to monitor the patients remotely. It may make the patients feel the loss of dignity and affect their quality of life in the long term [55]. In addition, AI applications have vulnerabilities that could be hacked or maliciously tampered with and produce unsafe outcomes [56]. In an adversarial attack case of medical AI, adding 4% adversarial noise to the original image caused AI to change the diagnosis of skin cancer. Whereas the image with superimposed noise was difficult for the human eye to distinguish compared to the original image, the benign diagnosis was incorrectly identified as malignant when the original image was rotated adversarially [57]. It suggests that a malicious hacker can deceive an AI system with subtle interference to harm humans. Therefore, whether algorithmic safety issues can be effectively addressed is related to whether medical AI can be widely adopted on a large scale. In addition, medical AI is still just a machine or program at this stage. It cannot adjust its actions to the actual situation and must rely on doctors to manipulate the machinery or make the final decision. At the beginning of its application, doctors may suffer from machine malfunctions due to inexperience and unskilled operation, some of which (e.g., operating errors in surgical robots) can have serious consequences. Patients would not necessarily have suffered this harm if not for the use of medical AI.

The data breach is also a security concern. In today's world, data is known as the "new oil" due to its economic value [58]. Healthcare data also has high research and business value, but data breach occurs frequently. 15% of global data breaches came from the healthcare industry in 2017, second only to the financial sector. The main situations of medical data breaches include cyber attacks such as hacker intrusion and unauthorized access, and even theft or loss of data by insiders in possession of the data. In 2020, a Chinese imaging AI was hacked and its source code and training data were sold on the dark web for 4 bitcoins (about 210,000 RMB) [59]. Much medical institution data is stored in the cloud or third-party servers, which is more vulnerable to hacking. The limited IT power of medical institutions also makes it difficult to ensure data security. The corresponding legal system has not yet been improved, resulting in the lack of effective regulation and restraint in medical data collection, use and privacy protection. Therefore, how to find a balance between medical data sharing and patient privacy protection is also an ethical problem faced in medical AI applications.

In addition, AI challenges the perceived authority of clinicians and may influence their independent judgment. Introducing AI into treatment decision-making may reintroduce the paternalistic model of “computer knows best”. Computers recommend treatments based on specific parameters that may not actually best reflect the values and preferences of a particular patient [60]. What is optimal for one patient in the same clinical situation may not be so for other patients. For example, most algorithms recommend treatment decisions based on which treatment best maximizes a patient's life expectancy; however, patients may prefer the treatment that minimizes pain. A machine learning algorithm may encounter ethical dilemmas if it is inconsistent with the doctor’s recommendations or does not consider the patient's inherent values and preferences. Thus, AI involvement may diminish the patient's subject position in clinical practices, undermine shared decision-making between doctors and patients, and threaten patient’s autonomy and dignity.

### Responsibility attribution

Medical AI replaces certain tasks previously performed by physicians, which will undoubtedly change the relationship between doctors and patients, and poses a dilemma in the division of ethical responsibilities. If medical accidents occur, who should be responsible? Can AI itself be the subject of liability? If not, what moral status should we give to AI? To what extent should it be held responsible? Or, who should be responsible for AI? These are all tough questions.

Before discussing whether AI is at fault, we need to clarify whether AI can be qualified as an independent legal responsibility subject. So, can AI be a subject of liability? The Turing test suggests that complex AI may have a certain level of consciousness [61]. In 2017, the humanoid robot Sophia, made by Hansen, was granted citizenship in Saudi Arabia, becoming the first robot to have its citizenship recognized. In the *Draft Report with Recommendations to the Commission on Civil Law Rules on Robotics *[62] the European Union has given intelligent devices the independent status of electronic persons with specific rights and obligations, making them eligible for liability for damage caused by themselves. It has introduced the rule that“a developer’s liability is inversely proportional to the autonomy of the AI robot” in the *European Civil Law Rules in Robotics* (ECLR), i.e., the more autonomous the robot, the less the trainer's liability. However, it is controversial to grant personality and rights to robots. Some scholars argue that although AI may surpass humans in many aspects, they do not possess free will essentially and does not have moral subjectivity [63, 64]. From Aristotle's “action must originate from the agent, and a person cannot be unaware of what he or she is doing”, AI neither meets the traditional criteria of free will nor is aware of what it is doing. Therefore, AI cannot be a responsible subject [65]. It is also the current consensus in the AI industry. Most scholars believe that AI robots at this stage can only belong to the category of tools yet. Current medical AI is only an auxiliary tool used by doctors to diagnose and treat diseases, not qualified to be a responsible subject. The medical AI Watson system passed the U.S. physician licensing exam in 2012 [66]. Although Watson is good at answering questions, it cannot independently and synthetically evaluate medical problems, nor can it independently conceptualize and search for new relevant information because it lacks the higher level of cognition required for critical thinking and the diversity of solutions ranked by the intrinsic value [67]. A gap still exists between the machine’s rule-based conditions and probabilistic statistics, and the human judgment mechanism, which is based on emotion and epiphanic meditation. Therefore, medical AI is still a tool in human medical activities at present, and humans should be responsible for AI.

For the emergence of strong AI or super AI in the future that people are worried about, its role may be closer to humans or even surpass humans, will AI at that time have the possibility of becoming a subject of responsibility? Some authors believe that in the context of strong AI, humans only play the role of a supervisor, and AI should be given the qualification of a legal subject [68]. Some who hold the opposite view believe that the subject-object sequential grid of natural law on the rules of obedience must be complied with. AI can only be framed in an objective class that cannot be personified [69]. We believe that the purpose of AI development is to serve humans better, not to subject humans to AI**.** The development of AI should not overly pursue some “technological singularity”. Technology should serve humans, not replace human talent and creativity. The autonomy of machines should not eliminate human subjectivity. Keep the development and application of AI technology from hindering human autonomy, and prevent the development and improper use of AI products beyond human control, which is the direction we should follow.

So who is responsible for AI applications? What kind of responsibility should they each bear? Who should be blamed if a doctor accepts a wrong diagnosis or treatment recommendation from a medical AI? Is it the doctor who makes the final decision, the medical institution that decides to use the AI, the producer of the AI, or the algorithm itself? What about when humans do not have enough control over the use of AI? In 2022, a report by the National Highway Traffic Safety Administration (NHTSA) showed that in an investigation of 16 Tesla crashes, several showed that Autopilot (Automated Assisted Driving) gave back control of the car to the human driver in "less than a second” on average before the crash and resulted in a total of 15 injuries and one fatality [70]. This automated setup means that even if the AI gives up control, none of the humans has time to intervene, so who is responsible for the accident? This scenario may also be seen in the automated decision-making of medical AI surgical robots. It can be seen that, due to the many stakeholders, we cannot simply blame the doctors in the application of medical AI, but need to bring in more responsible stakeholders, such as developers of medical AI, designers of algorithms, providers of training data, regulators of AI, etc.

## Discussion

**Fig. 2:**
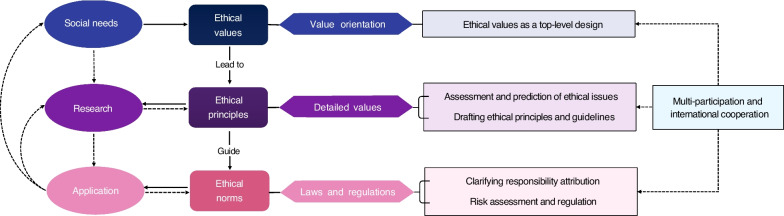
Ethical governance system

A new technology is first generated by social needs. Then people will conduct relevant scientific and ethical research. After that, we will put it into the application, continuously improve the technology and ethics in use to finally adapt the technology to the needs of society and ethics in line with the general values of people. It is the same for medical AI. For this new technology, we should fully assess the existing risks and those that may occur, which is analyzed earlier in the paper. To address the related ethical issues, we sorted out the governance countermeasures of trustworthy medical AI using the ethical governance framework of ethical values-ethical principles-ethical regulations (Fig. 2). Ethical values reflect the universal shared values of society. It is the orientation and top-level design for technology development and use. Ethical principles are the specific refinement of ethical values, which are the assessment and prediction of related risks from the perspectives of technology and ethics, and to guide the formulation of laws and regulations. In a nutshell, ethical values lead to ethical principles, and ethical principles guide ethical regulations. Therefore, combining the above affecting factors, we proposed the corresponding governance countermeasures for trustworthy medical AI from the ethical, legal and regulatory aspects.

### Ethical values as a top-level design

The law is a mandatory norm with certain lagging defects, and the legislative process is characterized by a harshness and long cycle, and more often than not, it can only be “hindsight” and cannot provide timely and effective protection measures. Therefore, ethics and morality become an effective complement to the legal system, and AI technology innovation must be carried out in accordance with ethical requirements. An ethical framework for AI design, manufacturing, and use should be established to evaluate the rights and wrongs of decisions and actions in the AI field. Ethical values as a foundation for developing AI technologies allows for broader presuppositions to address potential technological risks. In 2016, the Institute of Electrical and Electronics Engineers (IEEE) released its first AI report-*Ethically Aligned Design: A Vision for Prioritizing Human Well-being with Artificial intelligence and Autonomous System (AI/AS) *[71]. Since then, a large number of ethical principles and guidelines have been published by various subjects, including international organizations, governments, enterprises and academic groups. For example, *Asilomar AI Principles *[72]*, Ethical Guidelines for Trustworthy AI* by European Union [73], *Next Generation AI Governance Principles-Developing Responsible AI* from China [74], and *Ethics and Governance of Artificial Intelligence for Health: WHO Guidance *[75], etc. The fundamental purpose is to regulate and constrain the development and application of AI technologies. The common value goal mentioned in these ethical principles is to put the interests of human beings at the forefront. That is to say, the development and use of AI must promote the good of mankind. In addition to following AI’s ethical principles, medical AI should also comply with medical ethics. Beauchamp T.L. and Childress J.F. first proposed four major principles of bioethics in their book *Principles of Biomedical Ethics*: respect for autonomy, nonmaleficence, beneficence, and justice[76] These four principles have become universally recognized as bioethical principles to guide medical and research decisions. The value orientation of AI ethics and medical ethics is the same, that is, to promote human health and well-being, and the bottom line is to do no harm to humans. Medical AI, as a technology, contains the values of the developer or designer; therefore, those involved have an ethical responsibility for AI products.

Although people agree with ethical values, there is a huge gap between ethical orientation and application. For example, is the design of collecting users' private data to provide better services to them consistent with promoting the good and not harming human beings? These issues require sufficient ethical discussions and make certain restrictions on the practices to allow technology to develop sustainably in a controlled manner. Another example is the programming of autonomous vehicles. Whether the response in the face of a sudden emergency is designed to avoid pedestrians who suddenly cross the road but may hit an obstacle and cause casualties to the occupants of the car or to protect the safety of the occupants but may harm innocent passers-by, which will also involve the classic ethical dilemma "trolley problem". In 2016, the product leader of Mercedes-Benz responded to such questions raised by the media with "protecting the occupants of the car in priority", which is understandable for the manufacturer, otherwise, who would go for a car that does not protect them? However, it was not a responsible decision which also caused an outcry. Because the consumer group of Mercedes-Benz cars belongs to the wealthy class, does this mean that wealthy people can make the final decisions, which is unfair to the poor. So this is not only an ethical issue but also relates to the social acceptance of the products, which needs to be discussed in depth. It has also been proposed that with the increase of AI autonomous decision-making capability, ethical algorithms should be embedded in the algorithmic system to increase the reliability and security of AI decisions. Three approaches were considered: a top-down approach, a bottom-up approach, and a hybrid approach [77]. The top-down approach essentially converts moral rules into mathematical symbols to express algorithms that can respond to all ethical issues with a set of ethical principles. In fact, humans have no consensus on ethical dilemmas like the "trolley problem". It is unlikely to generate an ethical standard that everyone agrees on to deal with the issues, which also poses an ethical challenge for moral algorithms. On the other hand, the bottom-up approach simulates biological evolution. It enables AI systems to generate universal ethical principles from specific ethical situations through machine learning and self-organization [78]. The hybrid approach combines the first two and is the current mainstream of AI algorithm design. However, in either approach, AI’s response to the causality dilemma remains inadequate, and the threat to human moral subjectivity by allowing AI to form universal ethical guidelines spontaneously is even more deadly. What will help us move forward in AI ethics may not be a roadmap for grand narratives but rather a sensitive and sustained argument for the morality of AI decisions in specific contexts.

### Clarifying responsibility attribution

Improving laws and regulations related to AI is the fundamental guarantee for the implementation of ethical reshaping. Only by clarifying responsibilities and providing norms at the legal level can ethical constraints be made practical and feasible. At present, there is no unified standardized quality standard, access system, evaluation system and guarantee system for the application of AI in the medical field, and the related policy and regulation system has not been completely established yet. In addition, the algorithms of medical AI are based on the pre-existing human experience. Medicine itself is potentially risky and uncertain. Therefore, no matter how scientific AI is, there is always the possibility of making mistakes. Whether existing laws and regulations are applicable to attribute responsibility for medical disputes caused by medical AI is an important issue at the legal and practical levels.

In the previous section, we have elaborated that existing medical AIs are not moral enablers and do not have the ability to think and make decisions independently and cannot be considered as duty bearers. Humans should be responsible for AI. In order to better use AI, we need to divide the responsibilities of different actors. First, we can examine whether doctors have operational errors when using AI. If the doctor has errors in operation, the doctor and the medical institution are responsible. AI robot’s participation in diagnosis and treatment is predicated on the approval of the medical institution where it is located. If a doctor causes damage, the medical institution can recover compensation from the doctor after taking responsibility. It is also necessary to review whether the medical institution has put in place training for doctors in AI use in order to evaluate the extent of their liability. In the second scenario, the doctor has no improper use of AI, and the AI itself is faulty. In this scenario, AI researchers, designers, and manufacturers' responsibilities must be divided based on the problematic aspects of AI, such as data labeling, program design, and product quality. At the same time, doctors are not exempt from liability because they are the main actors in diagnosing patients. At the current level of medical AI development, doctors are still in the position of supervising and they should not let machines make final decisions without their permission. Besides, current medical AI falls under the category of medical devices, and both the department that approves AI for marketing and the medical institution that introduces AI in clinics need to consider whether there are loopholes in the process and risk control. In the third scenario, the related people are scrupulous in their duties, but still cannot prevent the medical AI from making an incorrect diagnosis that leads to the patients’ misfortune. There is no clear evidence of who is responsible, or we cannot attribute responsibility to any individual. That means there may be an empty field of responsibility. Floridi proposed a principle of moral responsibility of faultless responsibility, which means that no one is at fault, but they are still responsible for it. Floridi suggested that we can develop a mechanism that moves away from concerning the intentions and perceptions of each individual agent, but instead, allows these agents to act as a network that shares risk and responsibility [79]. However, this distributed responsibility may result in a lack of individual responsibility, leading to a tendency for everyone to be conservative and making the application and innovation of new technologies impossible. We can learn from the experience of Europe and the United States to add specific liability fees to the selling price of AI and try to establish a mandatory government or industry-led insurance and reserve system, with multiple parties such as developers, manufacturers, owners (medical institutions), and the government paying for the fees, and establish an independent pool of funds dedicated to the payment of legal liability for medical AI, so that both patients’ rights and interests can be effectively protected and prevent relevant subjects from losing the incentive to develop and use the technology due to the huge risks of liability. On the one hand, guiding provisions should be made in the existing laws to guide the healthy development of AI. On the other hand, attempts can be made to promote AI legislation at different levels, starting with more specific local and experimental legislation to provide experience for the AI legislative process.

### Risk assessment and regulation

Legal experts concerned with AI governance issues criticize ethical principles as flawed and inadequate in addressing AI’s ethical and social issues. A few companies are keen to propose ethical standards rather than binding rules. The reasons for this are apparent because there is no substantial penalty if they change or disregard ethical standards under this circumstance [80]. The most important job for ethicists is to clarify and elucidate the connotation of ethical principles and help scientific and technical workers to realize the transformation of ethical principles from macro to micro. In other words, ethicists should not only tell researchers what they should do but also assist them in solving more specific and detailed problems. Therefore, under the guidance of ethical principles, there is a need to develop more specific and operational guidelines and recommendations and translate ethical research results into governmental regulations or departmental rules so that ethical principles can have legal and administrative effects. Relevant subjects, including science and technology enterprises and workers in research institutions and industrial fields, should identify, prevent and manage risks through a strict risk management system and clarify the risk control responsibilities of each subject. The following regulatory directions are proposed to address the factors affecting AI trust presented in the previous sections.

#### Strengthening data management

Current AI technology essentially obtains data by measuring the real world, extracts algorithmic models from the data, and uses the models to make relevant predictions. Therefore, data and algorithms are the basis of AI computing and decision-making. The utilization rate of big data in healthcare is low. Although the data in hospitals are enormous, most of them are unstructured data, which cannot bring out the value of “big data”. Many hospitals have not yet established a unified data management system, which is not conducive to the unified analysis of data and impacts the application of AI technology in the medical field. Many countries have incorporated quality management of training data and data trainers into their regulatory frameworks to ensure data quality. For example, China’s *Deep Learning Assisted Decision-Making Medical Device Software Approval Points *[81] requires quality control of training data, and should ensure diversity of data sources, with data collected from multiple medical institutions at different geographic and hierarchical levels whenever possible. The *Approval points* further subdivide the data sets into training sets (for algorithm training), validation set (for algorithm hyperparameter tuning), and testing set (for algorithm performance evaluation), etc., and specifies different acquisition requirements. It also provides requirements for the access qualification, selection, training, and assessment of data trainers.

Second, on the sharing of health care data. The main obstacle to data sharing is the ownership of data. There are several views of data ownership in academic circles: ownership by individuals, ownership by organizations such as enterprises, ownership by the state, and ownership by all human beings. The debate around ownership does not only include questions as to who owns data, but also whether there should be a notion of ownership. Macnish and Gauttier [82] argue that it's not appropriate to talk about our relationship with data in terms of ownership. There are only weak philosophical grounds on giving citizens control of ‘their’ data. Control should be based around custody of data and the potential for harm. Healthcare data are sensitive information about a person [23, 83] which is also related to personal privacy. Respect for personal privacy is a crucial ethical principle in health care because privacy is linked to personal identity and autonomy [84]. For these reasons, proper procedures to ensure that genuine informed consent is obtained from patients regarding the use of their personal health data are essential. For example, patients must give explicit consent for their health data to be used for any specific purpose [85]. In 2018, the EU introduced the first bill on personal data privacy protection-*General Data Protection Regulation* (GDPR) [86]. Unlike previous industry regulations, this is a truly enforceable law with specific and strict requirements. For example, operators are required to allow users to express a desire for personal data to be “forgotten”, i.e., “I don’t want you to remember my past data and I want you not to use my data for modeling purposes from now on”. At the same time, the consequences of violating GDPR are severe, and fines can be as high as 4% of the global revenue of the fined organization. In practice, however, if software development organizations were to require patient consent for each use of aggregated data, it would inevitably increase the cost of data use. Manson and O’Neill [87] argue that more specific consent is not always ethically better and is difficult to achieve in practice. Consent requires unique communicative transactions. Through these communicative transactions, other obligations, prohibitions, and rights can be waived or set aside in a controlled or specific manner. Some scholars proposed more lenient forms of informed consent, such as broad consent and blanket consent, to facilitate practical implementation [88, 89]. However, the moral rationality for these informed consents remains controversial. Regarding the sharing of health care data, some believe that patients have an obligation to contribute to improving the quality of the health care system [90]. Patients’ clinical data have potential medical value and should be widely shared to promote the health and well-being of all humans. From the perspective of human benefits, it is also unethical not to use existing clinical data to develop tools to benefit all humanity [91]. In the author's view, health data should be applied rationally in the public interest while protecting patient privacy and data security. De-identification and anonymization can be used to protect patient privacy in data collection and storage. De-identification is the process of making it impossible to identify the subject’s personal information without the help of additional information by appropriate processing. For example, the identity information is represented by one-to-one unrelated code names, the AI software developers have access only to the code names, and the database owner holds the key to associate the code names with the identity. At the same time, the decoding must be stipulated accordingly. The anonymization process means that the personal identifiers in the data are completely removed and there is no connection between the data provider and its data. Anonymous data means that it cannot be used to identify a person and is therefore not subject to the GDPR rules, which means that if a company collects anonymous data, it does not need to obtain the consent of the users. Technologists also use differential privacy to create a barrier between hackers and data to prevent data from being restored after a breach [92]. We believe that it is ethical to dispense with re-informed consent for data use under conditions that ensure data security and do not compromise patient privacy, as long as a sound ethical review system is in place. If possible, the government should establish a corresponding website or query platform to facilitate patients to track their medical data usage status. A balance needs to be found between the two extremes: prohibit data flow for personal interests and pursue data sharing by putting public interests above personal interests. While ensuring medical data security, data sharing and research should reasonably be promoted to enhance human welfare, which is also the ethical and legal goal. On the premise of personal information protection, accessible data flow and strengthening international cooperation should be promoted through the United Nations, G20 and other global platforms to achieve sustainable development of AI.

#### Reducing algorithmic bias and increasing transparency and traceability

Reducing AI bias is necessary to promote better and more equitable health outcomes. To avoid bias, the design goal should be “ethics by design, not after a product has been designed and tested” [17]. AI manufacturers must be aware of the types of bias in medical AI and attempt to mitigate bias early in their product development process, such as identifying and minimizing the downstream impact of biased training datasets and cultivating technology developers in ethical literacy. Second, there is limited transparency in the black box of algorithms, whose inherent logic is hidden even from developers, and the lack of transparency may reduce the credibility of AI medical devices. Therefore, GDPR requires algorithms to have interpretability, and data subjects can take intervention and require interpretation of the relevant data when they are not satisfied with automated decisions. In fact, clinicians are also not always able to explain their inferences perfectly, as they may make decisions based more on experience and intuition than on clear medical criteria. Many of the drugs used in clinics may not be fully understood initially. For example, aspirin was used for about 70 years for its antipyretic, analgesic, and anti-inflammatory clinical effects, but its pharmacological mechanisms were not understood until later [93, 94]. Therefore, some believe doctors may be able to use some black box models in clinical practice as long as there is sufficient evidence that these models are reliable [95]. Interpretability is not a necessary or sufficient condition for accountability.

When algorithm explanation becomes more and more complex, we should appropriately turn our concerns to algorithm transparency and traceability. It is generally accepted that algorithm transparency means that algorithm developers should disclose the algorithm elements including source code, input data and output results. Most scholars believe that some degree of algorithmic transparency should be guaranteed by law, and various international documents also stipulate the principle of algorithmic transparency, such as *Ethics Guidelines for Trustworthy AI* issued by the European Union (EU), *Principles for Responsible Stewardship of Trustworthy AI* proposed by the G20, etc. Although algorithm transparency is not equal to algorithm explainability, it will form a powerful deterrent and encourage more diverse subjects, such as medical institutions, insurance companies, and social security institutions, to participate in supervision, which will greatly compensate for the lack of supervision of regulatory authorities. Some scholars suggest that disclosure of algorithm source code to relevant subjects be set as a legal obligation for companies to improve the post-marketing regulatory system of medical AI [96]. Nevertheless, algorithm transparency should also be coordinated with national security, social security, commercial secrets and other interests in an orderly manner, and build a “scenario-based algorithm transparency” with strict limits on the objects and contents disclosed by the algorithm. Algorithm traceability generally refers to the decision-making process of AI that should be fully recorded for future verification. In a sense, algorithm traceability is an extension of algorithm transparency, with the latter emphasizing static coding transparency and the former emphasizing dynamic algorithm operation transparency. In short, algorithmic transparency and traceability do not require algorithms to be explainable, but they provide the possibility of algorithmic explanation and form effective supervision. Humans may not have to fully explain AI for the time being, but we should create conditions and ensure that humans can explain AI in the future.

#### Whole-process review and supervision

We have explained AI's current lag in laws and regulations above. As a precursor and effective supplement to laws, the ethical review should run through the whole process of the design and use of AI. The risks and benefits of AI products should be thoroughly assessed and supervised by relevant organizations. First, the government should establish an AI ethics committee to oversee the direction of AI development and make corresponding changes and additions to previous systems, rules or laws and regulations based on supervision, inspection and evaluation results. All companies should review and approve the design and manufacture of robots through the relevant institutional ethics review committees, and programs with serious risks should be further ethically justified and reviewed and approved by higher-level ethics committees to ensure that their risk-to-benefit ratios and respect for people meet the requirements of ethical principles.

Secondly, medical AI will belong to the category of medical devices for a considerable period of time, and its main function is to assist doctors in diagnosis and treatment. Therefore, medical AI should be placed in the framework of medical devices for regulation. As of 2020, the U.S. Food and Drug Administration (FDA) has approved a total of 222 AI medical device products, and Europe has approved a total of 240 AI medical device products with European conformity certification [97]. Countries generally require or encourage medical device applicants to submit appropriate scientific research evidence to explain the scientific process and verify the safety and efficacy of the device at all stages of registration-including premarket approval and postmarketing studies.

Third, algorithms may be continually updated beyond their initially approved clinical function, which may require particular policies and supervision. Regulatory agencies must develop standard procedures, including effective post-sales monitoring mechanisms through which developers can document the development of their AI medical device products [83]. Educating users and patients about medical AI is also a way to ensure that they understand the benefits, risks, and limitations of medical AI devices and increase product transparency and user trust [98].

### Multi-participation and international cooperation

The challenges and risks facing medical AI are multifaceted, wide-ranging, and cross-fertilized. Therefore, the governance of healthcare AI requires the cohesion of multiple parties, including governments, professional communities, research institutions, healthcare facilities, the public, and the media. The professional community includes AI experts, medical experts, ethicists, and legal experts. All parties need to assess medical AI’s risks and social impacts before, during, and after the AI application.

The government should research and collect multiple opinions before formulating policies and laws. In the past, scientific and technological work was often the result of scientists setting up projects, the relevant departments or enterprises giving money, the government approving them, the public unaccountably affected, and humanities and social science experts cleaning up the mess. In fact, what needs to be done first is to involve humanities and social science experts upstream in the decision-making process, and to understand the background and results of the research. Experts from other disciplines, such as social sciences, law, and ethics should be brought in to collaborate so as to understand the attitudes of non-scientist groups and the possible ethical, legal, and social consequences of the work. The government should attract public representatives to participate in decision-making and establish monitoring and feedback channels. The professional community should try to propose and reach a consensus on ethical norms and governance of medical AI through adequate discussions and form industry norms. Technicians should strengthen ethical self-discipline and reflect ethical value orientation in the process of research and development. Many scientists already attach great importance to the ethical issues of AI, but it is still essential to strengthen relevant training and education. Doctors should also be involved in the research and development process of medical AI to improve the medical literacy of AI developers and the AI literacy of doctors. Trust in AI will improve through a more transparent development process with a better understanding of algorithms and AI functions. For AI companies, the vital thing should be to take social responsibility and take effective measures to prevent ethical risks rather than unilaterally pursuing economic interests. Only with the participation of all relevant sectors of society and multiple parties can an ethical and publicly acceptable medical AI be developed.

The challenges posed by medical AI are global, and its value goal is based on the fundamental interests of all human beings. Therefore, it is necessary to strengthen international cooperation and communication. However, international cooperation also faces many obstacles, such as cultural and legal systems differences in each country, which may lead to different attitudes and positions in the face of medical AI. Through sufficient discussions and communications, we can distill common themes and differentiated expressions, and establish a sound ethical governance system for medical AI that meets the actual situation of each country by taking into account its own conditions and drawing on advanced foreign experiences.

## Conclusion

In this paper, we explored the factors that affect the trustworthiness of medical AI, including poor data quality, algorithmic bias, opacity, safety and security risks, and difficulty in responsibility attribution. We proposed that ethical values should first be considered to guide AI development, with the promotion of human health and well-being as the fundamental goal. At the legal level, we clarified that medical AI does not have moral status at this stage, and humans remain the responsibility bearers. We tried to improve AI legislation by clarifying the attribution of relevant responsibilities based on existing laws. At the level of specific risk management, we proposed relevant countermeasures such as strengthening data quality management, data security, and privacy protection, promoting data set sharing, increasing algorithm transparency and traceability to reduce algorithm bias, and regulating and reviewing the whole process of AI, including design, production, marketing, and after-sales. Multiple parties should also be encouraged to participate in discussing and assessing AI risks and social impacts, and strengthen international cooperation and communication to address related challenges jointly.

## Data Availability

All data generated or analyzed during this study are included in this published article.

## Abbreviations

AI: Artificial intelligence
TCM: Traditional Chinese medicine
NLP: Natural language processing
XAI: Explainable artificial intelligence
FDA: Food and Drug Administration
ECLR: European Civil Law Rules in Robotics
NHTSA: The National Highway Traffic Safety Administration
IEEE: The Institute of Electrical and Electronics Engineers
AI/AS: Ethically aligned design: a vision for prioritizing human well-being with artificial intelligence and autonomous system
GDPR: General data protection regulation
EU: European Union
